# Effect of immersive virtual reality on pain in different dental procedures in children: a randomized controlled clinical trial

**DOI:** 10.3389/froh.2025.1539018

**Published:** 2025-09-17

**Authors:** Sabha Mahmoud Alshatrat, Jumana M. Sabarini, Hanan M. Hammouri, Majd M. Alsaleh, Isra Abdulkarim Al-Bakri, Abedelmalek Kalefh Tabnjh

**Affiliations:** 1 Department of Applied Dental Sciences, College of Applied Medical Sciences, Jordan University of Science and Technology, Irbid, Jordan; 2 Consultant of Pediatric Dentistry, Private Dental Center, Irbid, Jordan; 3 Department of Mathematics and Statistics, Faculty of Arts and Science, Jordan University of Science and Technology, Irbid, Jordan; 4 Department of Pediatric Dentistry, University of Illinois Chicago, Chicago, IL, United States; 5 Department of Cariology, Institute of Odontology, The Sahlgrenska Academy, University of Gothenburg, Gothenburg, Sweden; 6 Dental Research Unit, Center for Global Health Research, Saveetha Medical College and Hospital, Saveetha Institute of Medical and Technical Sciences, Saveetha University, Chennai, India

**Keywords:** virtual reality, dental pain perception, pediatric dentistry, local anesthesia, behavioral scales

## Abstract

**Introduction:**

Effective pain management techniques are fundamental for enhancing patients' adherence to various pediatric dental procedures.

**Objective:**

This study aims to evaluate the effectiveness of virtual reality (VR) on pain perception during dental procedures in children.

**Methodology:**

Children aged between 5 and 12 years were recruited from a pediatric dental clinic. Ethical approval and informed consent were obtained from all parents or guardians. The need for different dental procedures was determined for each child based on the outcome of a clinical examination. Some of these procedures require local anesthesia (LA) administration, while others do not. The children were randomly assigned to either a VR or a non-VR group. Three behavioral scales, the Visual Analogue Scale (VAS), the Wong-Baker FACES Pain Rating Scale, and the “Face, Legs, Activity, Cry, and Consolability” (FLACC) Scale, were used to assess the pain level during dental procedures.

**Results:**

A total of 154 children were recruited and evenly divided into VR and non-VR groups (77 each). The results of the study indicated that utilizing VR during dental procedures resulted in a significant reduction in pain perception (*p* < 0.05) and doubled the level of relaxation experienced by patients (70.31%), irrespective of anesthesia requirements. Statistical analysis revealed a significant difference between the VR and anesthesia groups across all VAS and Wong-Baker FACES variables.

**Conclusion:**

This research has confirmed that using virtual reality (VR) as a distraction technique effectively reduces pain during dental procedures for children. VR is a safe, non-invasive, and user-friendly technique that has gained interest as a non-pharmacological option for pain management. As a result, this promising approach has the potential to be used in clinical practice and should be further researched.

## 1 Introduction

Many people find dental procedures intimidating due to fears of pain or anxiety. Dental fear and anxiety can lead to serious oral health issues, making it a significant challenge in clinical dentistry (1). While dental fear and anxiety affect patients of all ages, it is more common among children and adolescents (2, 3). Due to this fear, many patients miss their dental appointments (4). The anticipation of pain or anxious thoughts can cause individuals to avoid dental treatment and neglect their regular dental care. Experiences—both positive and negative—can shape attitudes and behaviors toward medical care. When patients have positive experiences at the dentist, they are more likely to seek preventive care, which can lead to lower overall costs (5).

Many individuals experience fear or anxiety regarding dental procedures, specifically, which can complicate their treatment (6). In particular, the use of needle injections for local anesthesia often heightens this fear and anxiety, leading to increased sensitivity to pain during dental procedures. As a result, it is considered essential to distract patients during these treatments (7).

Pediatric dental offices must implement appropriate pain management techniques during treatment to ensure patient comfort, cooperation, and attendance, ultimately to maintain healthy teeth (8). There are two main categories of behavior management techniques: pharmacological and non-pharmacological. Pharmacological techniques include sedation and general anesthesia, while non-pharmacological techniques (also referred to as basic behavior guidance) encompass communication guidance, positive pre-visit imagery, direct observation, tell-show-do, ask-tell-ask, voice control, non-verbal communication, positive reinforcement, descriptive praise, desensitization, and distraction (9).

In dental offices, examples of distraction techniques are conversation, television, soap bubbles, counting, creativity in clinic design, and video games (10). Among these, distraction effectively reduces pain as well as fear and anxiety. Distraction techniques aim to redirect a patient's focus from unpleasant stimuli, potentially resulting in a less uncomfortable procedure (9).

Furthermore, passive and active are two types of distraction techniques. Passive techniques can be reading a book, listening to a story, or watching a video, requiring the patient to stay calm during the procedure. During dental procedures, patients can participate in active distraction techniques involving multiple sensory components, such as singing, controlled breathing, and using electronic devices (11, 12). Studies have demonstrated that distraction during procedures can significantly lower staff requirements, reduce procedural time, and minimize patient sedation. Moreover, it is a highly cost-effective alternative to analgesia (13).

Virtual reality (VR) has emerged as a novel, immersive form of active distraction that engages multiple senses to create a computer-generated environment. This technique effectively redirects attention away from painful or anxiety-inducing stimuli and has been shown to alleviate discomfort in various medical settings (14). Initially introduced for pain relief during burn wound care, VR demonstrated significant efficacy in reducing pain and anxiety during physical therapy (15, 16).

In pediatric dentistry, although research is still emerging, VR is gaining attention as a promising tool to manage pain and anxiety. Several recent studies have demonstrated its effectiveness in reducing discomfort during dental procedures among children (7, 15–19). For instance, Eijlers et al. conducted a systematic review and meta-analysis confirming VR's potential to reduce pain and anxiety in pediatric healthcare settings (17). Atzori et al. found that VR significantly lowered both anticipatory anxiety and reported pain in children undergoing dental treatment (20). Furthermore, Ran et al. observed physiological improvements such as reduced heart rate and shorter procedure durations in children using VR (21).

The underlying mechanisms of VR's analgesic effects are believed to involve attentional modulation and altered activity in the brain's pain-processing regions. Functional imaging studies suggest that VR reduces activation in the pain matrix while enhancing activity in areas like the anterior cingulate cortex and orbitofrontal cortex, which are associated with emotional and cognitive control (22, 23).

Despite this growing body of evidence, VR remains underutilized in dental settings, especially in low- and middle-income countries**.** In Jordan, there are limited studies investigating the effectiveness of VR in pain management during dental procedures for children (24). Moreover, most existing studies do not differentiate between types of dental procedures or account for special populations such as children with special healthcare needs (SHCN).

The gap in existing literature, particularly the limited studies on VR use in Jordan across different pediatric dental treatments, is the key issue our research seeks to address. By evaluating the effectiveness of VR in pain management for children undergoing various dental procedures in this context, we aim to provide valuable insights into its potential benefits and feasibility for this population.

## 2 Materials and methods

Ethical approval was obtained from the institutional review board (Ref: 64/124/2019).Our clinical trial was conducted in full adherence to the CONSORT (Consolidated Standards of Reporting Trials) checklist and guidelines for designing, conducting, and reporting randomized controlled trials. In line with international standards for clinical research, the trial has been registered with ClinicalTrials.gov (Clinical Trial ID: NCT06794788). The study was conducted per the Declaration of the World Medical Association. Per the Declaration of Helsinki, informed consent was obtained from all parents or guardians before including their children in the study. The research team conducted a pilot study to refine the methodology of the current clinical trial, provide the study team with experience in implementing the methodology, and confirm an appropriate sample size (19). The participants were recruited from a private pediatric dental clinic in Irbid, Jordan, over 12 months, from June 2023 to August 2024.

The baseline behavior of participants was assessed using the Frankl Behavior Rating Scale (FBRS), a widely used tool in pediatric dentistry for evaluating children's behavior during dental visits.21 This study only included participants with Frankl behavior ratings of 2 (negative cooperative) or 3 (positive). These ratings were assigned by the pediatric dentist after a thorough assessment during the initial evaluation prior to treatment. Including participants with these behavior ratings was crucial because children in these categories are more likely to respond to behavioral management techniques, such as distraction, which was the focus of our study.

Inclusion criteria for participation were children aged 5–12 years, in good health, taking no medications, and willing to participate in the study. Furthermore, children with Frankl behavior ratings of two (negative cooperative) or three were included in the study, ensuring no significant behavioral biases between the groups at baseline. Exclusion criteria included patients or children with a convulsive disorder, a history of severe vestibular abnormalities, and musculoskeletal or developmental delay in taking psychotropic drugs. Children with a Frankl behavior rating of 1 (definitely negative) were excluded from the study. This group typically exhibits severe dental anxiety—characterized by uncooperative behavior, intense fear, crying, or physical resistance—that often necessitates advanced behavior management techniques such as sedation or general anesthesia (9). Since this study focused on non-pharmacological distraction methods, specifically immersive virtual reality (VR), including these participants could have confounded the results. Their extreme anxiety and need for pharmacological intervention may have masked the effectiveness of VR, while their unpredictable responses could introduce significant variability and reduce internal validity.

Similarly, children with a Frankl behavior rating of 4 (definitely positive) were also excluded. These children are generally calm, cooperative, and show minimal or no signs of dental anxiety, making them unlikely to benefit meaningfully from a behavioral intervention. Their inclusion could have diluted the observed effect of VR, limiting the study's ability to detect measurable improvements. By focusing on children with Frankl ratings of 2 (negative) and 3 (positive)—who present with moderate levels of anxiety and cooperation—the study aimed to evaluate the effectiveness of immersive VR in a population where behavioral distraction techniques are most relevant.

Also, we included patients with prior dental experiences, especially positive ones. We recognize that prior exposure to dental procedures could indeed impact a child's anxiety levels and response to behavior management techniques.

The first dental visit should aim to create a positive, stress-free experience, applying the tell-show-do technique so children feel more comfortable returning for future visits. Thus, the use of VR goggles at a first dental visit may be inappropriate, as the technology would prevent the child from witnessing and becoming comfortable with the surrounding clinical environment.

All assessors experienced a comprehensive training program on properly using the assessment tools employed in this study, including those for measuring pain and behavior, to minimize bias and increase the reliability of those measurements.

### 2.1 Immersive virtual reality device, materials and procedures

**Figure 1:**
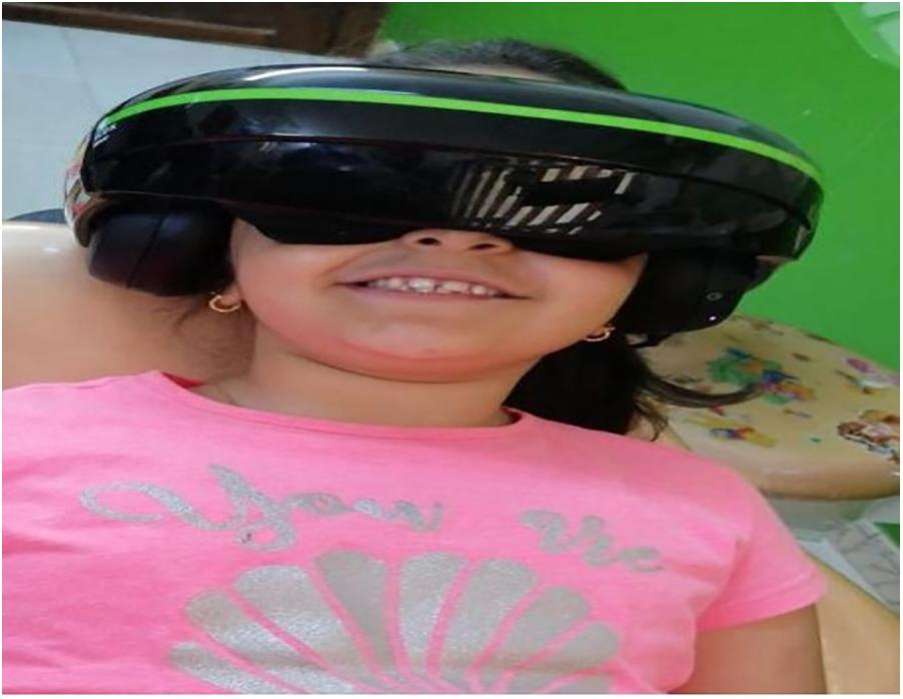
Immersive virtual reality as a distraction.

The virtual reality device used in this study is the iWear from Vuzix® in Rochester, New York, USA. It consists of lightweight, high-end video headphones with VR goggles (Figure 1).

Popular videos like Tom and Jerry, Mr. Bean, and children's songs in Arabic or English were used to create a virtual reality environment suitable for children. The children were allowed to choose the type of videos they wanted to watch before starting the procedure. The environment was designed to be non-violent and appropriate for pediatric patients. A personal computer was used to generate the VR environment.

**Figure 2:**
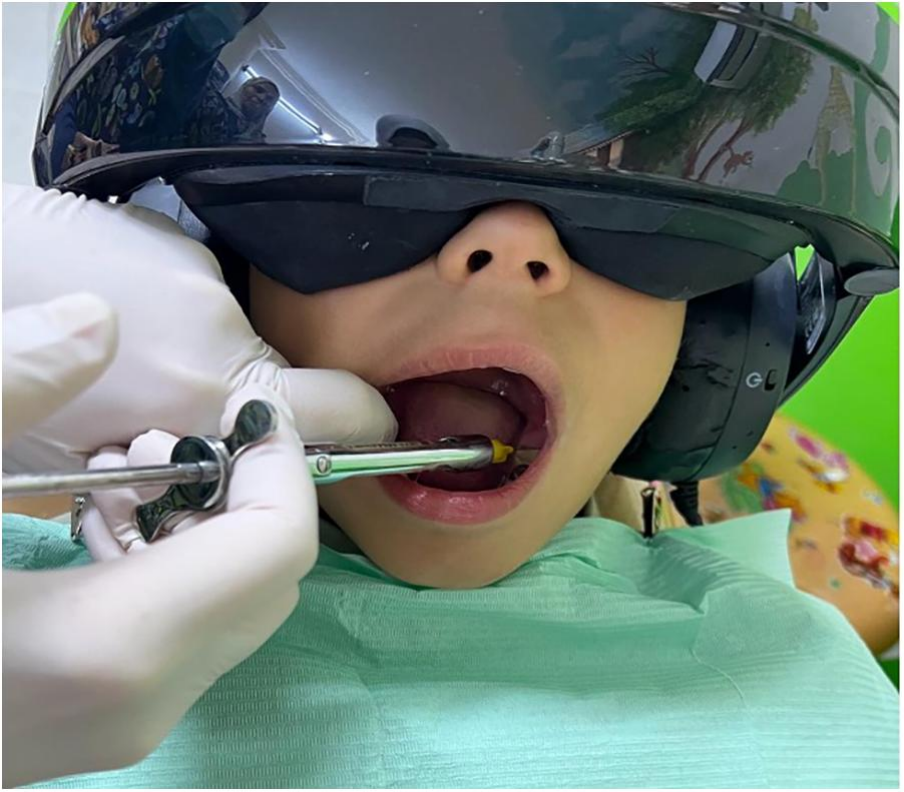
Local anaesthesia administration with immersive virtual reality.

A pediatric dentist assessed each child's treatment needs. A comprehensive oral examination of the entire mouth was conducted during the screening. Dental procedures were determined based on clinical examination. Some did not require local anesthesia, like fissure sealant, space maintainer, fluoride therapy, impression taking, and scaling. Local anesthesia is required for other dental procedures like stainless steel crowns, pulp therapy, restorations, and extractions (Figure 2). The pediatric dentist divided the children into two groups based on whether or not they needed local anesthesia (LA) for their dental procedures. Children not requiring LA were assigned to Group A, while those requiring LA were assigned to Group B. Then, a computer-generated random number sequence was used to randomly assign children in both Group A and Group B into two subgroups each, based on whether they received immersive virtual reality (VR) distraction during their dental procedures: Group A1 (no VR), Group A2 (VR), Group B1 (no VR), and Group B2 (VR).

**Figure 3:**
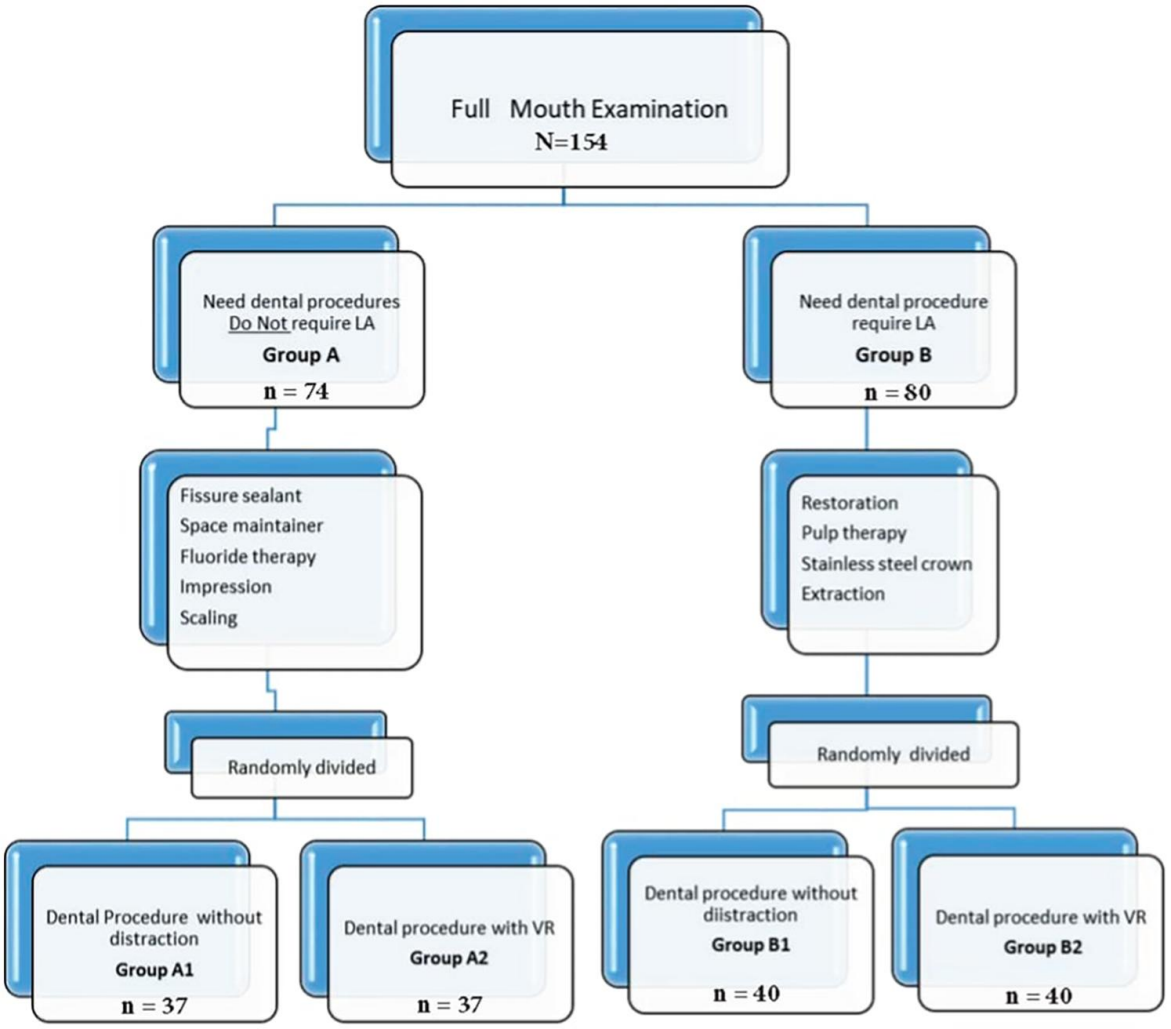
Flow diagram of the study design.

Using a computer-generated random number sequence ensured the random and unbiased allocation of participants (Figure 3).

Furthermore, to increase the validity of the randomization process, allocation concealment was implemented to prevent any foreknowledge of group assignments. Allocation concealment was achieved by using sealed, opaque envelopes containing the group assignment, which were only opened after participants were enrolled and had completed baseline assessments. This approach concealed the allocation from investigators and participants until the point of the assignment, preventing any potential bias in the selection process and maintaining the integrity of the randomization procedure.

The local anesthesia was administered via nerve block in the lower arch to ensure consistency and minimize potential confounding factors. Lidocaine 2% with epinephrine 1:100,000 solution was used for any inferior alveolar nerve block LA injection. For this study, a single pediatric dentist treated all children.

### 2.2 Pain assessment scales and data collection

#### 2.2.1 Wong-Baker FACES pain rating scale and the visual analogue scale (VAS), a self-report measure

**Figure 4:**
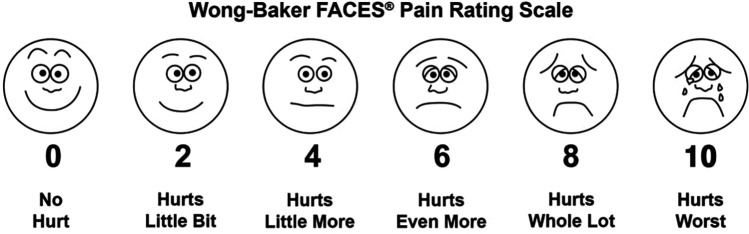
Wong Baker faces pain rating scale. Reproduced with permission from Wong-Baker FACES® Pain Rating Scale"

**Figure 5:**
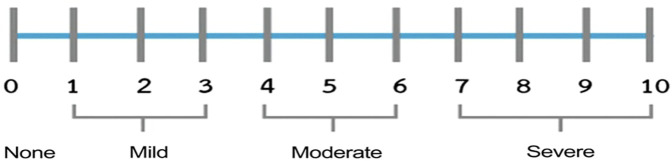
Visual analogue scale (VAS).

The study used two different pain measures, the Wong-Baker FACES Pain Rating Scale (Figure 4) and the Visual Analogue Scale VAS (Figure 5), to determine which one is easier for pediatric patients to understand and report during dental procedures. The Wong-Baker FACES Pain Rating Scale and Visual Analogue Scale (VAS) are commonly used and reliable tools for measuring pain in pediatric patients. The Wong-Baker FACES Pain Rating Scale is a pain assessment tool that uses six cartoon faces with varying expressions. The scale ranges from 0 to 10, where the patient selects the face that best represents their pain level.

The VAS is a self-report measure with a 10-centimeter horizontal line indicating the pain level. The children were asked to give ratings using 0–10 scales, with lower numbers indicating less pain while higher numbers indicating higher pain.

During the procedure, the participants were asked to rate how much time they spent thinking about their pain, how unfavorable dental care was, how much their teeth/gums caused discomfort, their worst pain, and average pain. Ratings for the Wong-Baker FACES Pain Scale and VAS were administered after treatment.

#### 2.2.2 Behavior assessment scale (external observation scale)

The “Face, Legs, Activity, Cry, and Consolability” (FLACC) Scale is a behavioral pain observation scale commonly used to measure pain during a procedure. The FLACC is a reliable and valid standard pain measurement tool used as an external evaluator to measure procedural pain (12, 19, 22, 25). The patient was evaluated in each of the five mentioned categories by an external evaluator who served as a research assistant. Each category is scored on a 0–2 scale, resulting in a total 0–10 score. The external evaluator used a 0–10 scale, with cut points indicating (0) Relaxed, (1–3) Mild discomfort, (4–6) Moderate pain, or (7–10) Severe discomfort/pain (25).

### 2.3 Data analyses

Descriptive statistics were used to summarize study variables. Means and standard deviations were calculated for continuous variables, while categorical variables were presented as counts (%). The association between FLACC, Wong-Baker FACES, and results was determined using Chi-square testing. A pairwise comparison of FLACC result levels between the two VR groups was conducted using Bonferroni adjustments and relative risk for significant results. Q–Q plots were used to check the normality of continuous outcomes. Multiple ANOVA tests were conducted to determine VR's effect in the presence of variables such as gender, anesthesia, and age. Statistical analyses were performed at a significant level of 0.05 using JMP software.

## 3 Results

A total of 154 patients who met the inclusion criteria participated in this study. The mean age of patients was 7.89 ± 1.96 years. The age distribution shows a significant difference between the Control and VR groups, indicating that the groups have slightly different average ages. Among the participants, 68 (44%) were males and 86 (56%) were females. However, there is no statistically significant difference in gender distribution between the groups, suggesting that the proportion of males and females is fairly balanced across the control and VR groups, with no significant difference in the distribution of children receiving anesthesia between the control and VR groups which suggests that the proportion of children receiving anesthesia is the same in both groups, and differences in the need for anesthesia do not bias the comparison between groups (Table 1).

**Table 1:** The descriptive statistics for the variables in the study are provided, along with the *p*-values.

Variable	Total	Control	VR	*P* value
Age	7.89 ± 1.96	7.56 ± 1.98	8.21 ± 1.89	0.0206
Gender				
Male	68 (44%)	32 (47%)	36 (53%)	0.5162*
Female	86 (56%)	45 (52%)	41 (48%)	
Anesthesia				
Yes	80 (52%)	40 (50%)	40 (50%)	1*
No	74 (48%)	37 (50%)	37 (50%)	

* For *P*-values, the chi-square test was used for categorical variables, and the *t*-test was used for continuous variables.

Means and standard deviations (SD) for the age variable and counts (%) for other variables.

One group of patients, Group A, received routine dental procedures without local anesthetic, while Group B received dental procedures that required local anesthetic, as shown in Figure 3. Table 1 depicts that the sample distribution was homogeneous. However, there was a noticeable age difference between the VR and non-VR groups. This difference was attributed to younger patients refusing to undergo VR, leading to a skewed distribution of age groups. Despite this, the study results were still considered reliable and informative, providing valuable insights into the effectiveness of VR technology in healthcare settings.

Our research evaluated the efficacy of virtual reality in reducing pain in children undergoing dental procedures with or without local anesthesia. The main aim was to test the hypothesis that employing virtual reality (VR) during dental treatment would help in reducing pain experienced by patients. The pain intensity was measured using two behavioral pain assessment scores, the FLACC and VAS scales and the Wong-Baker pain scale, which helped patients self-assess the severity of pain they felt during the treatment.

The statistical analysis revealed a significant correlation between the use of VR and the FLACC results (*P*-value = 0.0003) using the chi-square test.

**Figure 6:**
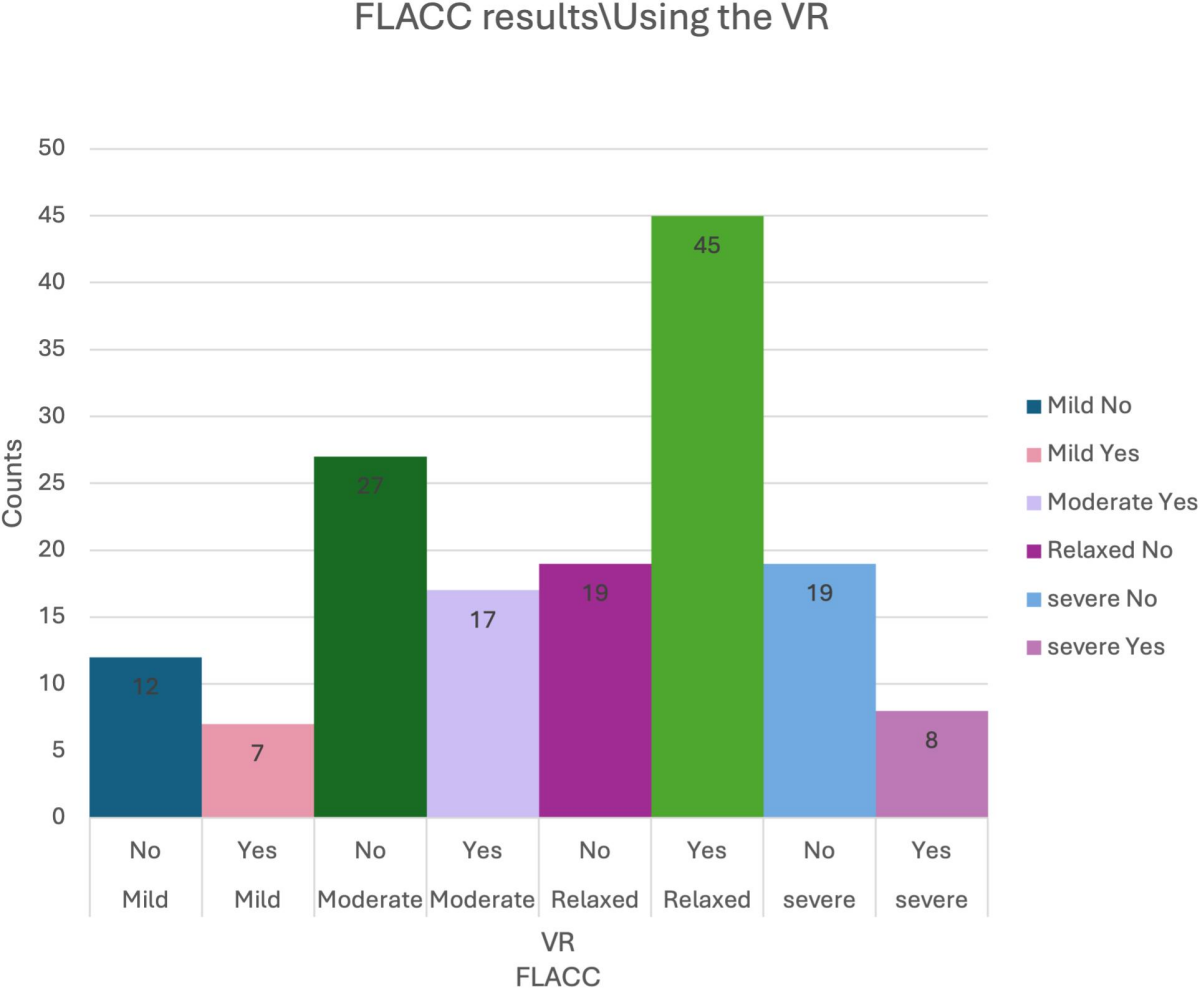
The counts for FLACC results for the two groups of VR.

To determine the underlying cause of this relationship, pairwise comparisons were conducted with Bonferroni adjustments. The results showed that patients who utilized VR were significantly more likely to be categorized as Relaxed (70.31%) compared to those categorized as Mild (36.84%), Moderate (38.64%), and Severe (29.63%). The results support the hypothesis that VR can be important in reducing pain and anxiety in pediatric dental settings (Table 2) (Figure 6).

**Table 2:** Counts and percentages for FLACC results for the two groups of VR.

FLACC results	Using VR		
	No	Yes	Total
Relaxed	19 (29.69%)	45 (70.31%)	19
Moderate	27 (61.36%)	17 (38.64%)	44
Mild	12 (63.16%)	7 (36.84%)	64
Severe	19 (70.37%)	8 (29.63%)	27
Total	77	77	154

On the other hand, it was found that anesthesia did not show significant interference in the VR group compared to the no-VR group. The results suggest that the use of VR had a significant effect on reducing pain in children, independent of whether anesthesia was used or not. Furthermore, the pairwise comparisons conducted using Bonferroni adjustment suggest that VR use was linked to a higher likelihood of children being in the “Relaxed” category, particularly when comparing “Relaxed” vs. “Mild,” “Moderate,” and “Severe” pain levels (Table 3).

**Table 3:** FLACC results levels were compared pairwise for the two VR groups using Bonferroni adjustment with the relative risk for significant results.

Levels	Relative risk	*P*-value bonferroni
(“Mild,” “Moderate”)		1.0000
(“Mild”, “Relaxed”)	2.1274	0.0485*
(“Mild”, “Severe”)		1.0000
(“Moderate”, “Relaxed”)	2.0670	0.0064*
(“Severe,” “Relaxed”)	2.3730	0.0019*
(“Severe”, “Moderate”)		1.0000

*Indicates statistically significant difference at *P* ≤ 0.05.

Q–Q plots were utilized to assess the normality assumption of continuous outcomes for VAS and Wong-Baker FACES variables. Based on the Q–Q plots, normality could be assumed, which is required for conducting ANOVA. Then, multiple ANOVA was conducted in the presence of variables such as gender, anesthesia, and age. All multiple-ANOVA variables were significant (the *p*-values for the whole models were significant), indicating differences in the VAS and Wong-Baker FACES variables of different groups (Table 4). VR and anesthesia significantly influenced pain and discomfort as measured by the Wong-Baker FACES and VAS scales.

**Table 4:** Parameter estimates and *P*-values for VR and anesthesia variables affecting Wong-Baker FACES and VAS scales.

Pain measure	VR			Anesthesia		
Term	Estimate	*t* Ratio	*P*-value	Estimate	*t* Ratio	*P*-value
Wong-Baker FACES	0.616	2.26	0.0250*	−1.272	−4.740	<.0001
Thinking of pain	0.767	2.90	0.0043*	−1.433	−5.480	<.0001
Discomfort	1.050	3.78	0.0002*	−1.279	−4.670	<.0001
Bothered	0.604	2.20	0.0295*	−1.067	−3.930	0.0001
Worst pain	0.728	2.57	0.0112*	−1.444	−5.170	<.0001
Average pain	0.575	2.10	0.0371*	−0.990	−3.660	0.0004

*Indicates statistically significant difference at *P* ≤ 0.05.

**Figure 7:**
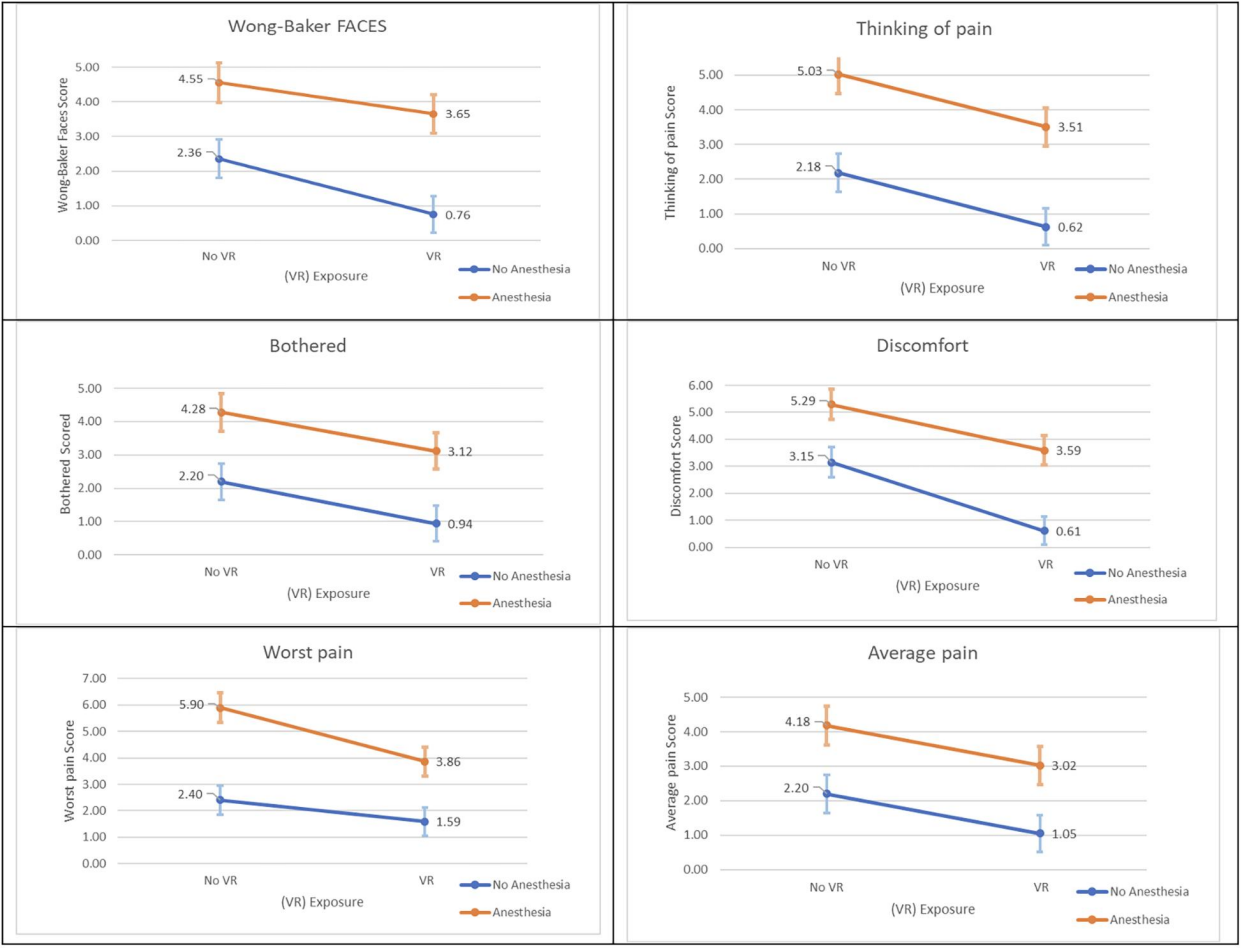
Wong-Baker FACES and VAS scores plots for means and SD for both VR groups by anesthesia groups.

All patients treated with VR had lower pain means than the control group, as shown in Figure 7, regardless of anesthesia used. The results show that the impact of virtual reality (VR) in reducing pain is consistent across different anesthesia groups. The statistical analysis demonstrated no significant interaction between the use of VR and anesthesia in all models. Therefore, the findings indicate that VR can effectively lower pain levels regardless of whether anesthesia is used.

## 4 Discussion

Dental fear and anxiety present significant challenges in pediatric dentistry, often preventing children from receiving essential dental care. However, a range of psychological and behavioral techniques has proven effective in modifying anxiety levels and the perception of pain during dental treatment (2, 3, 22). Notably, evidence indicates a strong correlation between pain-related anxiety and pain perception, which supports the use of validated pain scales in this study not only to measure pain experience but also as a proxy measure for related anxiety (25). Pediatric dentists commonly employ behavior management strategies to improve patient cooperation, reduce distress, and enhance treatment outcomes. Among these, conservative, non-pharmacological methods—such as distraction—are generally preferred over pharmacological alternatives due to ease of use and fewer resource demands. Unfortunately, barriers such as limited access to sedation services or anesthesiologists may impede the use of pharmacological techniques in some clinical settings (25).

Immersive virtual reality (VR) leverages multisensory engagement to divert attention from anxiety-provoking stimuli. This immersive distraction has demonstrated positive effects in altering both behavior and pain perception (9, 18, 26). McCaul and Mallet's attention-based theory (22) offers a framework for understanding VR's efficacy: humans possess a limited capacity for attention, and diverting this attention from painful stimuli to engaging environments—like VR—reduces the cognitive resources available for processing pain, thus decreasing perceived discomfort.

Further neurophysiological mechanisms have been proposed to explain the analgesic effects of VR. In particular, VR may influence the brain's pain signaling pathways by engaging areas related to attention, emotion, and memory (23). Functional imaging studies, including fMRI, have shown decreased activation in the pain matrix during VR exposure, alongside increased activity in areas such as the anterior cingulate cortex and orbitofrontal cortex—regions linked to emotional and cognitive modulation of pain. These findings suggest that VR may alter the brain's response to pain through top-down processes, ultimately reducing pain perception (23). Virtual reality has emerged as a promising behavioral management tool in pediatric dentistry, primarily due to its ability to block anxiety-triggering external stimuli and enhance child cooperation (27). As a distraction technique, VR is simple, non-invasive, requires minimal training, and has received broad acceptance from both parents and children (28, 29).

This study investigated the effectiveness of immersive VR distraction in managing pain among systemically healthy pediatric patients undergoing dental procedures, with or without local anesthesia (LA). The rationale for focusing on this population was to ensure internal consistency and eliminate confounding variables associated with systemic medical conditions, which could affect pain perception and behavioral response. While our results are encouraging, it is important to note that children with special healthcare needs (SHCN) often present with more complex behavioral and sensory profiles, which may influence the utility and effectiveness of audiovisual distraction techniques, including VR. Future research should explore adaptations of VR technology for SHCN populations, as the current evidence base is limited in this regard. Incorporating VR into this context holds potential, but may require tailored interventions and additional safeguards to accommodate specific medical and psychological needs, which represents a critical next step in extending the benefits of VR to more vulnerable pediatric populations.

Our study builds upon a previous pilot trial by the same authors involving 54 participants, which demonstrated the effectiveness of VR distraction in reducing dental anxiety and pain. In this larger study, 154 children were randomized into control and VR groups. Results confirmed that VR distraction significantly reduced pain perception, as measured by the FLACC scale, regardless of whether local anesthesia was administered. These findings align with the pilot data and reinforce the utility of VR as an effective behavioral management tool in pediatric dentistry.

Children in the VR group consistently demonstrated lower FLACC scores during treatment, with statistically significant differences compared to the control group. Consistent with prior findings, pain levels during LA injection were significantly reduced when VR audiovisual distraction was used (30). Ran et al. similarly reported that VR use was associated with reductions in physiological markers of anxiety (e.g., heart rate, oxygen saturation) and shortened treatment duration (21). While our study did not include physiological measurements, the alignment in subjective scales such as FLACC adds strength to these findings**.** We employed validated subjective measures—VAS, Wong-Baker Faces Scale, and FLACC—to evaluate pain perception. These scales revealed a consistent advantage for the VR groups across both anesthetized and non-anesthetized subgroups. Our results mirror those of Atzori et al., who found that VR distraction was effective in reducing pain perception among children and adolescents undergoing medical procedures (20).

Studies evaluating audiovisual eyeglass distraction have reported similar findings, demonstrating reductions in both anxiety and pain during conventional dental treatments, consistent with our results (31–34). For example, Koticha et al. observed improved physiological parameters in children using VR distraction, though no significant changes were found in self-reported anxiety on the Venham Picture Test (33). Felemban et al. also noted no statistically significant differences in pain scores between VR and control groups when LA was administered, though mean scores were lower in the VR group (34). Discrepancies with our results may be attributed to differences in study design, sample size (which was notably smaller in the Felemban study), or methods of pain assessment.

Importantly, VR should not be seen as a replacement for traditional behavioral techniques but as a complementary tool that enhances patient comfort, minimizes anxiety, and reduces the perceived need for sedation or general anesthesia. Its simplicity, non-invasiveness, and acceptability make it an attractive option in both clinical and resource-limited settings.

While this study primarily focused on pain perception—measured via VAS and Wong-Baker scales—we acknowledge the complex interplay between anxiety and pain. Studies have consistently shown that pain-related anxiety can heighten pain perception, thus supporting the use of pain scores as proxy measures for anxiety in pediatric dental research (35).

Limitations of this study include the limited immersive capacity of the VR headset (field of view: 55°, resolution: 1,280 × 768 pixels), which may have reduced the full immersive potential. A second limitation concerns the age differences between the VR and non-VR groups. Although the differences in FLACC scores were statistically significant (*p* = 0.0206), clinical relevance should be interpreted cautiously. Furthermore, baseline exposure to VR was not assessed, which may influence individual responsiveness to the intervention. Another important limitation is the lack of follow-up to assess longer-term effects, such as whether VR distraction influences future dental anxiety, behavior, or willingness to return for treatment.

The study's strengths include its robust sample size, adherence to a standardized protocol based on the pilot study, and consistency in procedural execution by a single dentist and assistant—minimizing inter-operator variability.

Future research should consider conducting subgroup analyses using narrower age bands to identify developmental differences in children's responses to VR. Additionally, procedure-specific outcomes should be examined to determine how different types of dental interventions influence the effectiveness of VR. The impact of first-time dental visits on children's behavioral responses to VR also should be examined. Longitudinal follow-up studies are necessary to assess both the immediate and long-term effects of VR interventions, providing a more comprehensive understanding of the impact over time. Furthermore, the incorporation of objective physiological measures (e.g., heart rate, blood pressure) can supplement subjective pain scores. Exploring the use of newer-generation VR devices and customized content tailored to age, language, and patient preferences could further enhance the intervention's effectiveness. Finally, future studies should include larger, more diverse populations across various cultural and clinical settings to enhance the generalizability of results.

Such studies will further refine our understanding of VR's role in pediatric dental care and support broader applications—including those involving children with special healthcare needs.

## 5 Conclusion

This study confirms that immersive virtual reality (VR) is a safe, effective, and well-accepted non-pharmacological tool for reducing pain in pediatric dental procedures. Significant reductions in pain were observed across FLACC, Wong-Baker FACES, and VAS scales, regardless of local anesthesia use. While limitations such as limited headset immersion and age-related variability exist, VR consistently enhanced patient comfort and cooperation. Future research should address physiological measures, prior VR exposure, and applications for children with special healthcare needs to expand its clinical relevance.

The findings of this study have important clinical implications for pediatric dentistry. Immersive virtual reality (VR) offers a safe, non-invasive, and engaging distraction technique that can be easily integrated into clinical practice to help manage procedural pain in children. For pediatric patients with moderate anxiety levels, who often respond well to behavioral interventions, VR may serve as an effective alternative or supplement to traditional techniques such as tell-show-do or audiovisual distraction. By improving the child's comfort and reducing perceived pain during treatment, VR may also enhance cooperation, reduce the need for pharmacological interventions, and contribute to more positive long-term attitudes toward dental care, which is particularly relevant in routine dental practices where managing anxiety and ensuring a positive treatment experience are essential for successful outcomes and patient retention.

## Data Availability

The raw data supporting the conclusions of this article will be made available by the authors, without undue reservation.
